# COVID-19 pandemic affects STEMI numbers and in-hospital mortality: results of a nationwide analysis in Germany

**DOI:** 10.1007/s00392-022-02102-2

**Published:** 2022-10-06

**Authors:** Vera Oettinger, Peter Stachon, Ingo Hilgendorf, Adrian Heidenreich, Manfred Zehender, Dirk Westermann, Klaus Kaier, Constantin von zur Mühlen

**Affiliations:** 1 Department of Cardiology and Angiology, University Heart Center, Faculty of Medicine, Medical Center, University of Freiburg, Hugstetter Str. 55, 79106 Freiburg, Germany; 2 Center for Big Data Analysis in Cardiology (CeBAC), Department of Cardiology and Angiology, University Heart Center, Faculty of Medicine, Medical Center, University of Freiburg, Freiburg, Germany; 3 Institute of Medical Biometry and Statistics, Faculty of Medicine and Medical Center, University of Freiburg, Freiburg, Germany

**Keywords:** COVID-19 pandemic, STEMI, Myocardial infarction, In-hospital mortality, National electronic health records

## Abstract

**Background:**

The COVID-19 pandemic led to extensive restrictions in Germany in 2020, including the postponement of elective interventions. We examined the impact on ST-elevation myocardial infarction (STEMI) as an acute and non-postponable disease.

**Methods:**

Using German national records, all STEMI between 2017 and 2020 were identified. Using the number of STEMI cases between 2017 and 2019, we created a forecast for 2020 and compared it with the observed number of STEMI in 2020.

**Results:**

From 2017 to 2020, 248,062 patients were treated for STEMI in Germany. Mean age was 65.21 years and 28.36% were female. When comparing forecasted and observed STEMI in 2020, a correlation can be seen: noticeable fewer STEMI were treated in those weeks respectively months with an increasing COVID-19 hospitalization rate (monthly percentage decrease in STEMI: March − 14.85%, April − 13.39%, November − 11.92%, December − 22.95%). At the same time, the crude in-hospital mortality after STEMI increased significantly at the peaks of the first and second waves (relative risk/RR of monthly in-hospital mortality: April RR = 1.11 [95% CI 1.02; 1.21], November RR = 1.13 [1.04; 1.24], December RR = 1.16 [1.06; 1.27]).

**Conclusion:**

The COVID-19 pandemic led to a noticeable decrease in the number of STEMI interventions in Germany at the peaks of the first and second waves in 2020, corresponding to an increase in COVID-19 hospitalizations. At the same time, in-hospital mortality after STEMI increased significantly in these phases.

**Graphical abstract:**

Impact of the COVID-19 pandemic on STEMI numbers and in-hospital mortality in Germany. Relative difference between forecasted and observed STEMI numbers (above figure), the relative risk of in-hospital mortality (middle figure) as well as number of new hospital admissions for COVID-19 per million inhabitants according to Roser et al.^27^ (bottom figure).

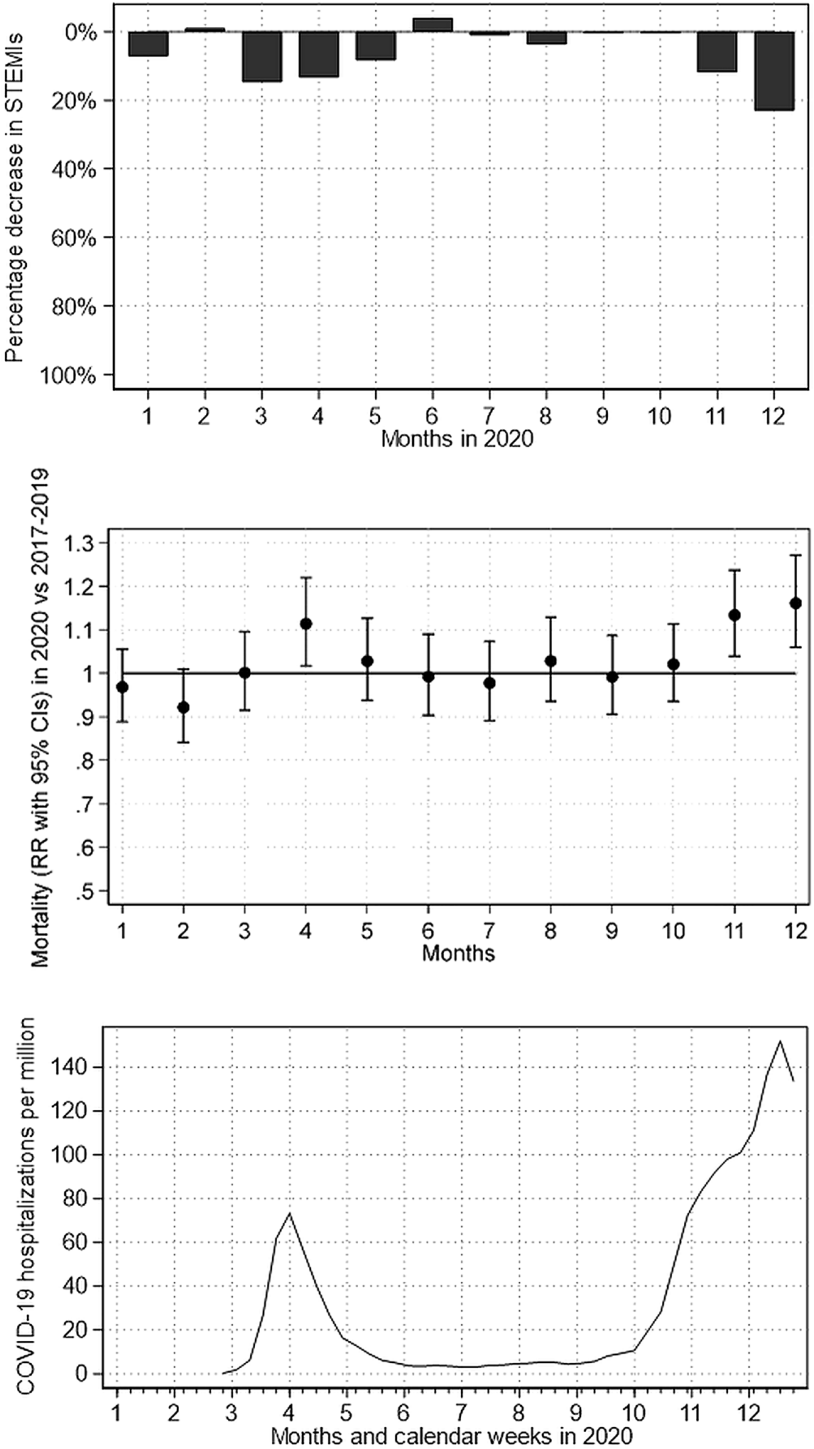

**Supplementary Information:**

The online version contains supplementary material available at 10.1007/s00392-022-02102-2.

## Introduction

In Germany, the first lockdown restrictions due to the upcoming COVID-19 pandemic were announced on March 16, 2020. This also had a strong impact on the German healthcare system. In order to maintain hospital resources for COVID-19 patients, the postponement of elective patients was recommended [1]. However, although emergencies continued to be treated, it is discussed in the international literature that even patients with actually life-threatening diseases might have refrained from hospitalization for fear of a potential SARS-CoV-2 infection. This is also applied in particular to cardiovascular diseases such as acute coronary syndrome or myocardial infarction [2–4].

Concerning ST-elevation myocardial infarction (STEMI), several studies in Germany [5–7] as well as internationally [2, 8, 9] have already confirmed the influence of the COVID-19 pandemic in the form of declining numbers of STEMI interventions, but their results regarding in-hospital mortality after STEMI since the beginning of the COVID-19 pandemic are still unclear. In addition, nationwide analyzes are still lacking.

Therefore, this study examines the impact of the COVID-19 pandemic on the number of catheterizations due to STEMI, using nationwide healthcare data. We compare forecasted and observed numbers of STEMI in 2020 as well as in-hospital mortality throughout the first and second waves of the COVID-19 pandemic. Finally, recommendations are to be made for future handling of the pandemic with regard to emergency interventions such as STEMI.

## Material and methods

The German Federal Statistical Office has been providing data on all hospital stays in Germany via its Research Data Center since 2005. The basis of these data is inpatient hospital billing under the German Diagnosis Related Groups (DRG) system, which is based on fixed charge groups—formed on the basis of diagnoses (coded according to ICD-10) and measures performed (coded according to the German Operation and Procedure Classification/OPS). Upon prespecified request, the Research Data Center may provide analysis of their data in the form of fully anonymous, aggregated results that are released by the Research Data Center. If necessary, partial results are censored. Our study did not involve direct access by the investigators to data on individual patients but only access to summary results provided by the Research Data Center. Therefore, approval by an ethics committee and informed consent were determined not to be required, in accordance with German law. All summary results were anonymized by Research Data Center. In practice, this means that any information allowing the drawing of conclusions about a single patient or a specific hospital was censored by Research Data Center to guarantee data protection. Moreover, to prevent the possibility to draw conclusions from a single hospital, the data are verified and situationally censored by Research Data Center in those cases.

This database represents a virtually complete collection of all hospitalizations in German hospitals that are reimbursed according to the DRG system. From this database, we extracted data on all STEMI (ICD-10 code I21.0, I21.1, I21.2 or I21.3) cases admitted to German hospitals between 2017 and 2020.

Using the number of STEMI cases between 2017 and 2019, we estimated the expected number of STEMI cases in 2020. In detail, this STEMI forecast was calculated by application of poisson regression models with the number of weekly (or monthly) STEMI procedures as endpoints and the calendar year and the calendar week (or month) as continuous and categorical covariates, respectively. These regression models included all procedures (2017–2019) and were then used for the prediction of the expected number of STEMI cases in 2020. Finally, the expected number of STEMI cases in 2020 is then compared to the actually observed number of STEMI cases in 2020. See Supplemental Table 1 for details of the two regression models. In the next step, crude monthly in-hospital STEMI mortality is compared between pre-pandemic (2017–2019) periods and 2020. Thereby, relative risks (RR) are calculated using poisson regression models with the application of robust standard errors.

When visualizing the results on a weekly basis, calendar weeks 53 and 1 are omitted due to problems in length. All analyses were carried out using Stata 16.0 (StataCorp, College Station, Texas, USA).

## Results

### Baseline characteristics and unadjusted endpoints

From 2017 to 2020, 248,062 patients were treated for a STEMI in Germany (Table 1). While 62,352–62,492 patients were treated per year from 2017 to 2019, in 2020 there were only 60,730 patients. Mean age was 65.21 years and 28.36% were female, which was comparable in all years observed. Other baseline characteristics such as logistic EuroSCORE and the extent of the coronary artery disease, i.e. 1‐, 2- or 3-vessel disease, were also comparable in all years.

**Table 1:** Baseline characteristics and unadjusted endpoints of all STEMI procedures in Germany in 2017–2020

	2017		2018		2019		2020		Total	
*N*	62,488		62,352		62,492		60,730		248,062	
Logistic EuroSCORE, mean/SD	7.25	7.23	7.34	7.31	7.33	7.27	7.39	7.34	7.33	7.29
Age in years, mean/SD	65.04	13.02	65.21	13.04	65.26	12.96	65.32	12.91	65.21	12.98
Female, %	28.89%		28.07%		28.32%		28.14%		28.36%	
1‐vessel CAD, %	29.64%		29.33%		29.53%		29.08%		29.40%	
2‐vessel CAD, %	28.49%		28.28%		28.32%		28.65%		28.43%	
3‐vessel CAD, %	36.63%		37.30%		37.24%		37.47%		37.16%	
Left main stenosis, %	6.10%		6.43%		6.18%		6.14%		6.21%	
Mortality, %	10.83%		10.96%		10.87%		11.09%		10.94%	
Stent implantation, %	86.85%		87.63%		88.01%		88.48%		87.73%	
Ventilation > 48 h, %	8.49%		8.32%		8.13%		8.42%		8.34%	

*CAD* coronary artery disease, *EuroSCORE* European System for Cardiac Operative Risk Evaluation, *N* number of procedures, *SD* standard deviation

In-hospital mortality at 11.09% in 2020 was only slightly higher than the average at 10.94%. Regarding stent implantations, a continuous increase from 2017 to 2020 from 86.85 to 88.48% can be observed. The rate of mechanical ventilation > 48 h was comparable in all years.

### Comparison of forecasted and observed STEMI in 2020

**Fig. 1:**
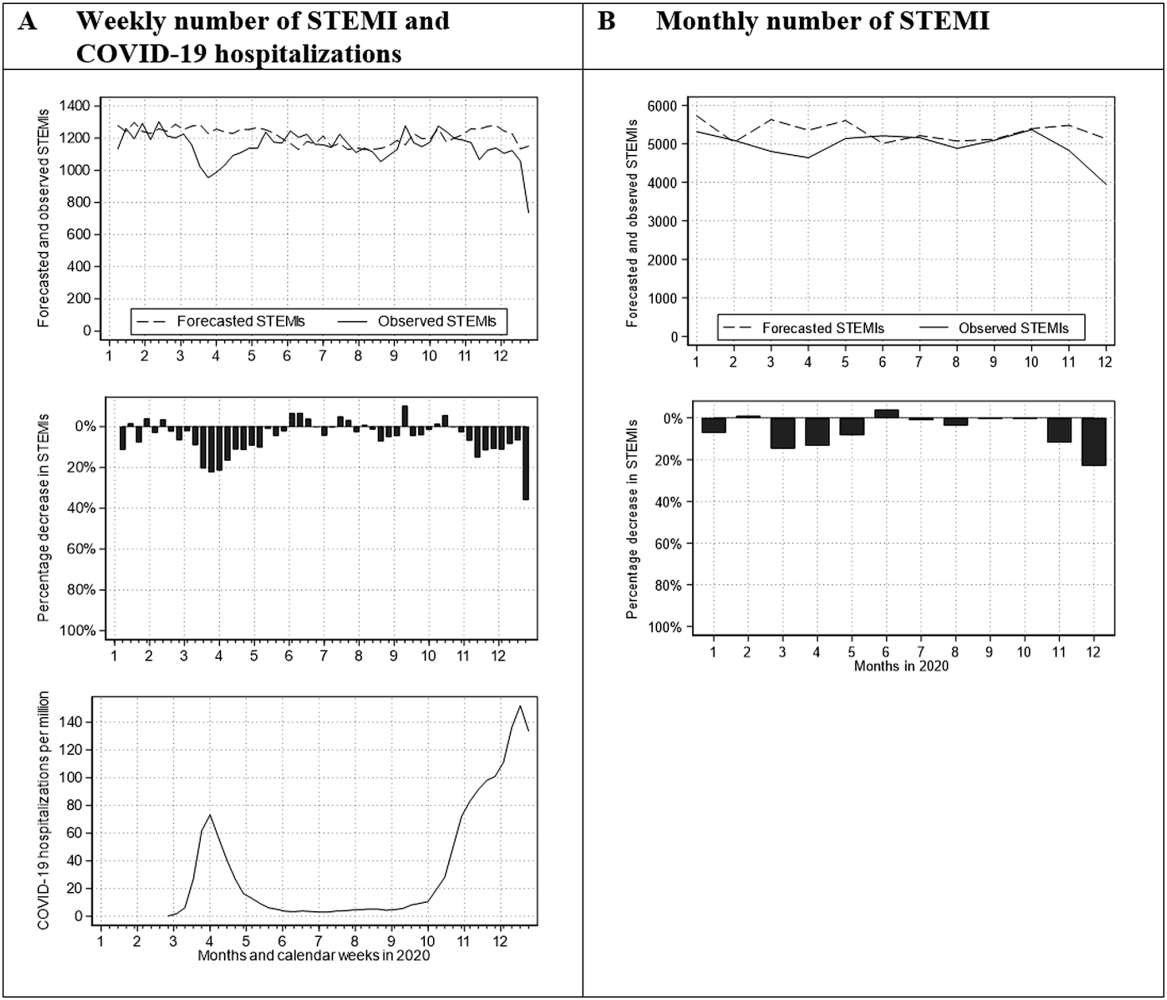
Weekly and monthly number of STEMI as well as weekly number of COVID-19 hospitalizations in Germany. Forecasted and observed weekly and monthly number of STEMI interventions (above figures in **A** and **B**), relative difference between forecasted and observed weekly and monthly number of STEMI interventions (middle figures in **A** and **B**) as well as weekly number of new hospital admissions for COVID-19 per million inhabitants according to Roser et al. [27] (bottom figure in **A**)

When comparing forecasted and observed STEMI numbers in 2020, a correlation can be seen (Fig. 1): noticeable fewer STEMI were treated in those weeks respectively months with an increasing COVID-19 hospitalization rate (monthly percentage decrease in STEMI: March − 14.85%, April − 13.39%, November − 11.92%, December − 22.95%).

However, the observed number of STEMI interventions in those months with only small COVID-19 hospitalization rates following the first wave reached the forecasted value but did not exceed it in response to the previous lower ones. This resulted in an overall reduction of STEMI interventions in 2020 compared to 2017–2019.

**Fig. 2:**
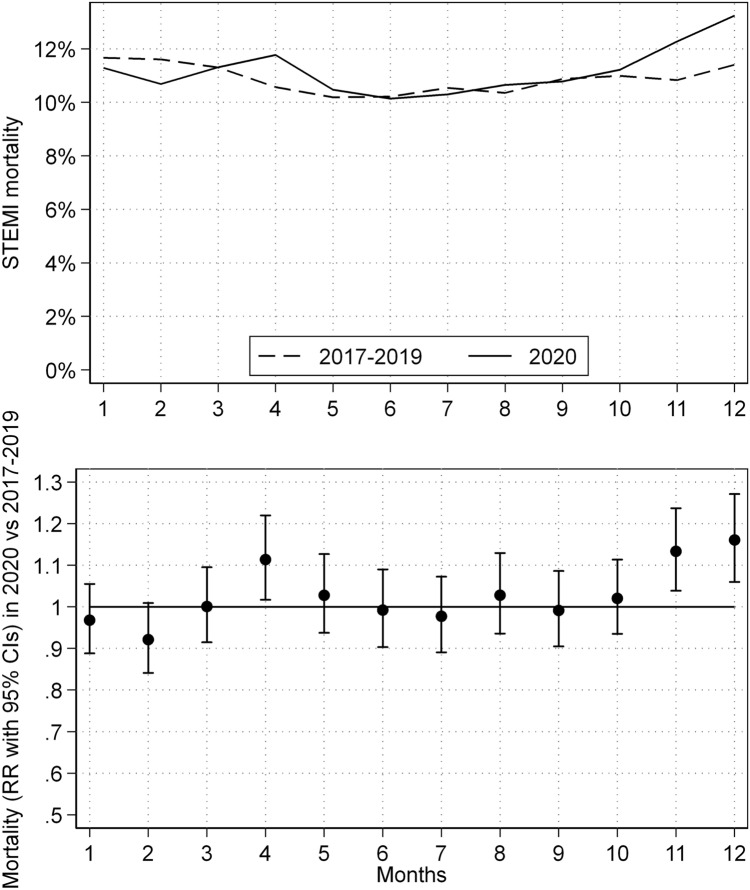
Monthly in-hospital mortality after STEMI intervention in Germany. Forecasted and observed monthly in-hospital mortality after STEMI (above figure) as well as the relative risk of monthly in-hospital mortality (bottom figure). *RR* relative risk; *CI* confidence interval

When considering the forecasted and observed monthly in-hospital mortality in case of STEMI (Fig. 2), crude in-hospital mortality after STEMI increased significantly at the peaks of the first and second waves in 2020 (monthly in-hospital mortality: April RR = 1.11 [95% CI 1.02; 1.21], November RR = 1.13 [1.04; 1.24], December RR = 1.16 [1.06; 1.27]).

## Discussion

Our results show that the COVID-19 pandemic resulted in a noticeable decrease in the number of STEMI interventions in Germany at the peaks of the first and second waves in 2020, corresponding to an increasing rate of COVID-19 hospitalizations. Meanwhile, in-hospital mortality after STEMI increased significantly during these phases.

Decreases in the number of STEMI procedures during the lockdown were also reported by Mesnier et al. [10] in a registry study in France. Furthermore, the authors noted that there was no catch-up phenomenon in the 4 weeks following the lockdown. But they analyzed only 8 weeks, the 4 weeks preceding and following the lockdown. However, we observed a whole year. Our study confirmed those results for the entire year 2020: No catch-up effect could be observed in those months with only small COVID-19 hospitalization rates following the first wave. This resulted in an overall reduction of STEMI in Germany in 2020. There are several possible explanations for the lower number of STEMI: The population may have moved less during the lockdown, so STEMI occurred less frequently due to a reduction of excessive physical activity. The same applies to a possible decrease of professional stress, e.g. by reducing travel times or working in a familiar environment at home [2, 10]. However, these effects are speculative since physical activity is generally also considered to be a protective factor for cardiovascular disease and all-cause mortality [11, 12]. Furthermore, previous studies suggest that the rate of depression and mental stress during the COVID-19 pandemic may even have increased [13]. In addition, less pollution may have contributed to the reduced STEMI numbers. Alternatively, patients with STEMI may not have contacted medical emergency services due to fear of a SARS-CoV-2 infection [2, 10, 14]. In the last case, a catch-up effect is not to be expected.

Similar results with declining patient numbers during the COVID-19 pandemic but higher in-hospital mortality have also been observed in other disciplines such as acute stroke care [15] or in patients undergoing cholecystectomy [16].

Furthermore, Rattka et al. [2] analyzed the topic in a meta-analysis of ten international studies with 50,123 STEMI patients. In contrast to our results, the authors found no increase in in-hospital mortality. They argue that information campaigns may have been quickly implemented and pre- as well as in-hospital procedures may have been rapidly optimized by the medical teams and cardiology professionals. However, they also point out that the observed period may have been too short to discover significant differences in mortality.

On the other hand, and in line with our results, Gluckman et al. [17] indicated an increasing in-hospital mortality after STEMI during the lockdown. They discussed a possible delay by patients, medical services or cardiac catheterization laboratories. Furthermore, Wienbergen et al. [18] showed a significantly higher rate of patients presenting with cardiogenic shock and out-of-hospital cardiac arrest. That may be associated with a delayed presentation in the case of STEMI and may also have contributed to the increased in-hospital mortality demonstrated in our study. Therefore, early hospital admission in the case of STEMI seems particularly important.

Consequently, people and especially patients with already known heart disease such as coronary artery disease must be made aware that they should report to medical emergency services in the event of acute symptoms, pointing out the high risk of mortality and morbidity in these cases.

The study has certain strengths and limitations, in accordance with previous analyzes [19–24]. A major strength is the use of a very large, complete national data set of all STEMI based on electronic health records, allowing us to draw conclusions with high applicability. There are also limitations beyond those typical of retrospective studies. The analysis relies on administrative data, so coding errors are almost unavoidable. Usually, however, 20% of DRG are reviewed by independent physician teams from health insurances, so overall reliability should be good. Furthermore, data is limited to the actual hospital stay as well as to the coded data that are transmitted by the hospitals. Therefore, statements about parameters before or after the stay, or a differentiation within the stay are not possible. This refers e.g. to parameters like rate of cardiogenic shocks or resuscitations before admission, time delays to hospitalization, or the door-to-balloon time. For the same reason, rates of out-of-hospital deaths or unsuccessful resuscitations before hospital admission due to STEMI could not be captured and thus the overall mortality may be even higher than the observed in-hospital mortality suggests. In addition, the use of risk scores recommended in the ESC Guidelines [25, 26], e.g. the GRACE risk score, was not suitable because it can hardly be calculated using the data sent to DESTATIS by the hospitals (e.g. no transmission of precise heart rate or blood pressure). The logistic EuroSCORE used in this study is therefore one way of at least roughly estimating the risk. Finally, no long-term follow-up is possible because the data source used does not allow a connection between different hospital stays of the same patient. Our study thus solely provides data on in-hospital outcomes, although for a very large, complete national yearly cohort of procedures.

## Conclusions

In summary, the COVID-19 pandemic led to a noticeable decrease in the number of STEMI interventions in Germany at the peaks of the first and second waves in 2020. At the same time, in-hospital mortality after STEMI increased significantly in these phases.

## Supplementary Information

Below is the link to the electronic supplementary material.

Supplementary file1 (PDF 35 KB)

## Data Availability

Data are available upon reasonable request. The patients’ data are stored on the server of the Federal Bureau of statistics and are not available due to data protection. The calculated raw data are sent anonymized to the scientist.
