# Supplementary material for: COVID-19 pandemic affects STEMI numbers and in-hospital mortality: results of a nationwide analysis in Germany
Source: Clin Res Cardiol. 2022 Oct 6;112(4):550–7. doi: 10.1007/s00392-022-02102-2 (PMC9534737; doi:10.1007/s00392-022-02102-2)
Supplement: Supplementary file 1 — Supplementary file1 (PDF 35 KB) [file 392_2022_2102_MOESM1_ESM.pdf]

Supplemental Table 1: Poisson regression models to predict the number of STEMI in 2020

Regression 2017-2019

		**Coeff**	**p-value**	**95%CI**	
year		0.01	0.343	-0.01	0.04
month					
	2	-0.13	0.000	-0.16	-0.10
	3	-0.02	0.433	-0.06	0.03
	4	-0.07	0.000	-0.10	-0.04
	5	-0.02	0.071	-0.05	0.00
	6	-0.14	0.000	-0.16	-0.11
	7	-0.10	0.000	-0.12	-0.07
	8	-0.12	0.000	-0.18	-0.07
	9	-0.11	0.000	-0.15	-0.08
	10	-0.06	0.000	-0.09	-0.03
	11	-0.04	0.000	-0.07	-0.02
	12	-0.11	0.066	-0.23	0.01
_cons		-17.15	0.528	-70.44	36.14

prediction for 2020

month		**Coeff**	**p-value**	**95%CI**	
	1	5735.98	0.000	5399.00	6072.97
	2	5033.83	0.000	4748.33	5319.33
	3	5637.05	0.000	5259.49	6014.62
	4	5354.85	0.000	5061.66	5648.04
	5	5610.44	0.000	5314.31	5906.57
	6	5003.01	0.000	4736.91	5269.12
	7	5210.82	0.000	4935.87	5485.77
	8	5065.98	0.000	4728.79	5403.17
	9	5113.20	0.000	4806.82	5419.59
	10	5394.40	0.000	5100.25	5688.55
	11	5483.77	0.000	5192.70	5774.83
	12	5125.344	0.000	4719.178	5531.509
